# A Comprehensive Structural Analysis of *Clostridium botulinum* Neurotoxin A Cell-Binding Domain from Different Subtypes

**DOI:** 10.3390/toxins15020092

**Published:** 2023-01-18

**Authors:** Kyle S. Gregory, K. Ravi Acharya

**Affiliations:** Department of Life Sciences, University of Bath, Claverton Down, Bath BA2 7AY, UK

**Keywords:** botulinum neurotoxin, cell-binding domain, ganglioside binding, synaptic vesicle glycoprotein 2, structural analysis

## Abstract

Botulinum neurotoxins (BoNTs) cause flaccid neuromuscular paralysis by cleaving one of the SNARE (soluble *N*-ethylmaleimide-sensitive factor attachment protein receptor) complex proteins. BoNTs display high affinity and specificity for neuromuscular junctions, making them one of the most potent neurotoxins known to date. There are seven serologically distinct BoNTs (serotypes BoNT/A to BoNT/G) which can be further divided into subtypes (e.g., BoNT/A1, BoNT/A2…) based on small changes in their amino acid sequence. Of these, BoNT/A1 and BoNT/B1 have been utilised to treat various diseases associated with spasticity and hypersecretion. There are potentially many more BoNT variants with differing toxicological profiles that may display other therapeutic benefits. This review is focused on the structural analysis of the cell-binding domain from BoNT/A1 to BoNT/A6 subtypes (H**_C_**/A1 to H**_C_**/A6), including features such as a ganglioside binding site (GBS), a dynamic loop, a synaptic vesicle glycoprotein 2 (SV2) binding site, a possible Lys–Cys/Cys–Cys bridge, and a hinge motion between the H_CN_ and H_CC_ subdomains. Characterising structural features across subtypes provides a better understanding of how the cell-binding domain functions and may aid the development of novel therapeutics.

## 1. Introduction

*Clostridium botulinum* neurotoxin (commonly referred to as BoNT) is widely utilised within the pharmaceutical industry to treat a range of diseases associated with muscular and endocrine overactivity. There are over 100 approved medicinal applications of BoNT, such as the treatment of spasticity, blepharospasm and dystonia [1], as well as for aesthetic indications [2].

Historically, all bacteria producing botulinum neurotoxin were classified as *Clostridium botulinum*, and this metabolically diverse species was divided into four distinct phenotypes (Groups I, II, III, and IV) [3]. BoNT can cause a potentially fatal disease, botulism, that is characterised by flaccid paralysis due to inhibition of acetylcholine neurotransmission. *C. botulinum* groups I (proteolytic) and II (non-proteolytic) are responsible for botulism in humans [4], whereas group III causes animal botulism [5,6] and *C. botulinum* group IV does not cause botulism. There are many BoNT variants which have been categorised into distinct serotypes due to neutralisation by different antibodies. A total of seven serotypes [7] (BoNT/A to /G) have been identified as well as a novel serotype (BoNT/X) that is not neutralised by any known BoNT antibody [8,9]. Advancements in genome sequencing have also led to the discovery of mosaic BoNTs (BoNT/FA(HA), /CD, and /DC) [10,11,12,13] as well as BoNT-like proteins from *Weissella oryzae* (‘BoNT/Wo’) [14,15,16], *Enterococcus faecium* (‘BoNT/En’) [17,18], and *Paraclostridium bifermentans* (‘PMP1’) [19]. Toxicity across serotypes varies significantly, with BoNT/A, /B, /E, and /F associated with human disease [20]. Each serotype within BoNT is further divided into subtypes (e.g., BoNT/A1-/A10, BoNT/B1-/B8, BoNT/E1-/E12, BoNT/F1-/F9) due to differences within their amino acid sequence [21]. The similarity cut-off for any newly identified BoNT being defined as a subtype has been arbitrarily set to >2.5% [22], meaning within the subtypes themselves there is additional variation. This huge diversity of BoNTs suggests the toxin may have a viral origin [23], and at the domain level subtypes appear chimeric as they have evolved at different rates [24]. Most subtypes, within the same serotype, bind the same antibody and cleave the same substrate at the same site; however, they display different binding characteristics to monoclonal and polyclonal antibodies [25,26,27,28]. One exception is BoNT/F5, which cleaves its substrate at a different site compared to the other BoNT/F subtypes [29]. Therefore, understanding the functional and structural differences across different subtypes is important for the development of novel BoNT-based therapies.

**Figure 1:**
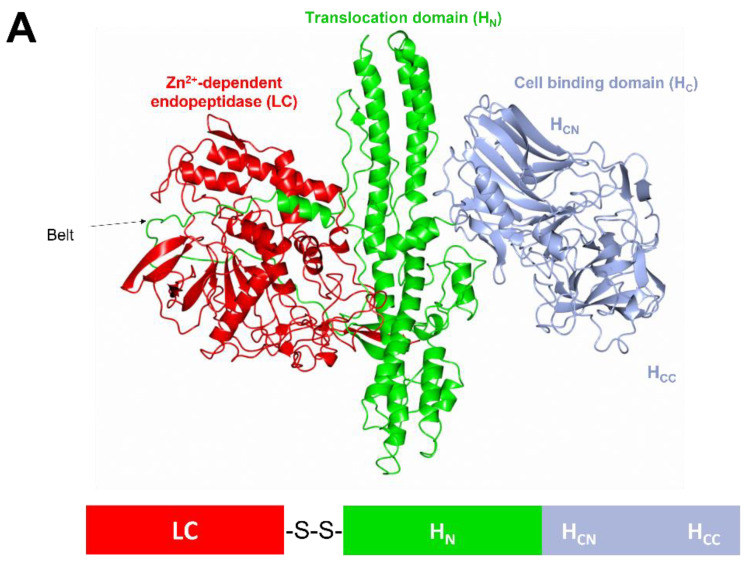

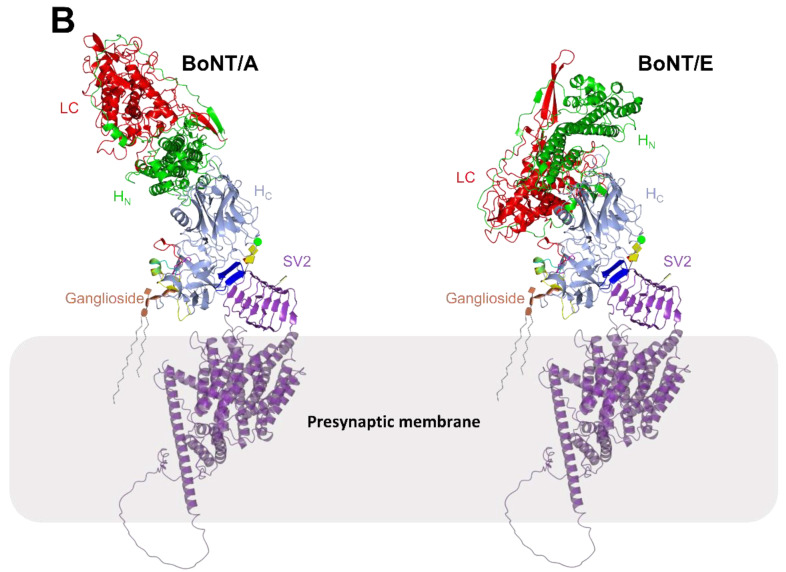
Structure of botulinum neurotoxin and a model of dual receptor binding at the neuromuscular junction. (**A**) Crystal structure of BoNT/A (PDB 3BTA) and schematic of linear protein. (**B**) Model of BoNT/A (PDB 3BTA) [30] and BoNT/E (PDB 3FFZ) [36] binding to a neuromuscular junction membrane by superimposition of BoNT/A and BoNT/E with the AlphaFold2 model of SV2C (synaptic vesicle glycoprotein 2 isoform ‘C’) (uniprot Q496J9) and the SV2C-bound structure of H_C_/A1 [37] (PDB 5JLV). The two models illustrate the different domain organisation between BoNT/A and BoNT/E, where the latter is thought to display enhanced translocation ability by positioning both the LC and H_N_ more closely to the membrane [36,38]. SV2C is coloured purple, the H_C_ domain grey, H_N_ domain green, LC red, and ganglioside orange.

The molecular structure of BoNT/A (Figure 1A) consists of both a light chain (LC) of ~50 kDa and a heavy chain (HC) of ~100 kDa that are held together by a single disulphide bond [30]. BoNTs are expressed as a single polypeptide chain that is cleaved post-translationally to form an active di-chain molecule by either a native or host protease [31,32]. The LC possesses a catalytic domain, a Zn^2+^-dependent endopeptidase, whereas the HC comprises two domains: a translocation domain (H_N_) and a cell-binding domain (H_C_). The H_N_ resembles a coiled-coil structure with a ~50 residue long ‘belt’ region that wraps around the LC [30] (Figure 1A) inhibiting endopeptidase activity by behaving as a pseudo-substrate [33]. The cell-binding domain contains an N-terminal β-jelly-roll fold and C-terminal β-trefoil fold which are referred to as the H_CN_ and H_CC_ subdomains, respectively [34] (Figure 1A). Most BoNT serotypes are thought to adopt a domain arrangement in what can be described as an ‘open-wing’ conformation based on the crystal structures of BoNT/A (Figure 1A) [30] and BoNT/B [35]. The exception to this is BoNT/E, which adopts a ‘closed-wing’ conformation [36] (Figure 1B).

**Figure 2:**
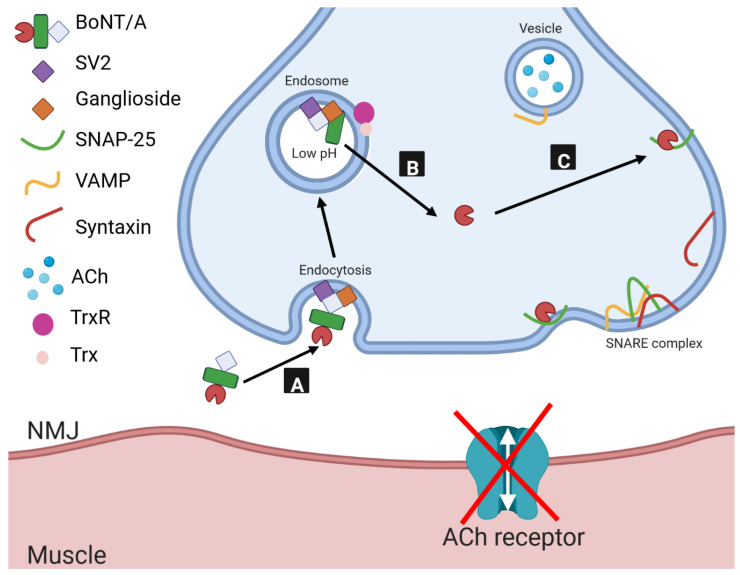
Modular mechanism of BoNT/A activity. (**A**) On arrival at the neuromuscular junction (NMJ), the cell-binding domain binds to both a ganglioside and SV2 on the surface of motor neurons where it is then internalised by receptor-mediated endocytosis. (**B**) The acidic environment of the endosome results in conformational change of the translocation domain which facilitates the passage of the LC through the endosomal membrane into the cytosol. Cytosolic thioredoxin (Trx) and thioredoxin reductase (TrxR) reduce the disulphide bond connecting the LC to the HC, releasing the LC into the cytosol. (**C**) The LC then cleaves its target SNARE protein (SNAP-25), preventing vesicular fusion and acetylcholine (ACh) secretion into the neuromuscular junction.

The three domains have specific functions that contribute to the mechanism of toxicity (Figure 2). The first step involves target-cell binding by the H_CC_ subdomain [39] upon arrival at the neuromuscular junction (NMJ) (Figure 2A). This subdomain (with the exception of BoNT/C1) binds to both a ganglioside and protein receptor present on the surface of motor neurons (Figure 1B) [40]. Two protein receptors have been identified for BoNT—three isoforms of synaptic vesicle glycoprotein 2 (SV2A-C) which are recognised by BoNT/A [41], BoNT/D [42], BoNT/E [43], and BoNT/F [44], and two isoforms of synaptotagmin (sytI-II) which are recognised by BoNT/B [45,46] and BoNT/G [47].

Following binding, BoNT is internalised through receptor-mediated endocytosis [48]. Subsequent acidification of the endosome causes a conformational change of the H_N_ domain and triggers translocation [49]. Although the precise molecular basis of translocation has not yet been elucidated, the conformational change to the H_N_ is widely believed to grant the passage of the LC through the endosomal membrane into the cytosol [37,38,49,50,51,52,53,54,55,56] (Figure 2B). Reduction of the disulphide bond connecting the LC and HC, by cytosolic thioredoxin (Trx) and thioredoxin reductase [57], is required for release of the LC from the membrane-bound HC. Upon release, the LC is then free to cleave its target SNARE (soluble *N*-ethylmaleimide-sensitive factor attachment protein receptor) protein (Figure 2C). SNARE proteins are involved in the formation of the SNARE complex that facilitates vesicular fusion and exocytosis of neurotransmitters and hormones from cells. BoNT/A, /C, and /E cleave the SNARE protein SNAP-25 [58], whereas BoNT/B, /D, /F, and /G cleave VAMP [59,60,61,62]; BoNT/C also cleaves syntaxin [63]. Cleavage of any one of these SNARE proteins at the NMJ prevents release of acetylcholine [64]. The precise mechanisms underpinning post-translocation trafficking of BoNT LC are yet to be established, however, recent evidence suggests LC/A1 is co-localised to the plasma membrane along with SNAP-25 [65].

BoNT/A is the most well-characterised serotype, with currently ten subtypes identified through a blast pairwise alignment [66]. The first eight subtypes vary in amino acid sequence between 3 and 16% [67]. Although the differences in amino acid sequence across BoNT/A subtypes are small, they have been associated with significant variation in toxicity. For BoNT subtypes /A1 to /A6, both cell entry rates and enzyme kinetics have been shown to differ, but the duration of action (measured by the time in which SNAP-25 hydrolysis in primary rat spinal cord cells exposed to BoNT stops) is due to the LC domain alone [68]. BoNT subtypes /A1, /A2, /A4, /A5, and /A6 action persists for >10 months, whereas /A3 lasts much shorter, only 3 months [69,70]. This long duration of action is thought to be partly due to the ubiquitin proteasome system, which removes ubiquitin from the LC inside neuronal cells, preventing ubiquitin-dependent protein degradation [71]. Although the duration of action is comparable across the subtypes (with the exception of A3), the extent of SNAP-25 hydrolysis, and therefore potency, has been shown to vary [72,73,74,75]. As the catalytic residues are 100% conserved, this difference in potency is likely driven by exosites within the LC or by other domains [67,76].

Compared to BoNT/A1, BoNT/A2 displays a similar potency in mouse models [77], however, it enters cells faster due to differences in both the H_N_ and H_C_ domains [38,77]. BoNT/A2 also has a higher affinity for gangliosides than BoNT/A1 [39] and remains more localised with faster onset of paralysis compared to BoNT subtypes /A1, /A3, /A4, and /A5 [78]. Although the potency of BoNT/A1 and BoNT/A2 are similar in vivo, the catalytic activity of BoNT/A2 in vitro is five times lower than that of BoNT/A1 [72]. Not only does BoNT/A3 display the shortest duration of action, it also has the lowest potency in cells, compared to the other subtypes [69,72,74]. Compared to BoNT/A1, both BoNT/A3 and BoNT/A4 display a 2-fold and 1000-fold lower activity in mouse models due to less efficient cell entry [69,72]. BoNT/A5 showed similar potency in mice but was less potent in human cell models compared to BoNT/A1 [72], suggesting a possible variance in activity within subtypes across species. For BoNT/A6, potency was higher than BoNT/A1 with faster entry kinetics, and like BoNT/A2 [69,72,78] the onset of SNAP-25 cleavage by BoNT/A6 occurred more rapidly than by /A1 in neurons [70]. However, unlike both BoNT/A1 and BoNT/A2, BoNT/A6 cleaved more SNAP-25 [70]. Considering that the LC activities of BoNT/A6 and BoNT/A1 in vitro are similar, the earlier onset of BoNT/A6 activity is likely due to more efficient cell entry [70]. However, it is interesting to note that, although BoNT/A2 and BoNT/A6 have a similar and increased potency compared to BoNT/A1 in rat and human cell models, they required a two times higher toxin dose upon local intramuscular injection to be lethal in mice [70,75]. Both BoNT/A7 and BoNT/A8 show similar lethal activity in mice compared to BoNT/A1 [79]; however, the latter was also shown to have reduced ganglioside binding and lower enzymatic activity in vitro compared to /A1 [80].

**Figure 3:**
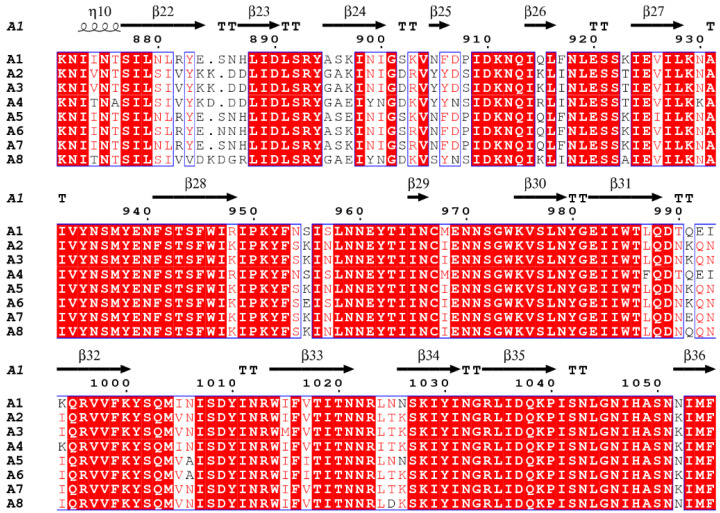

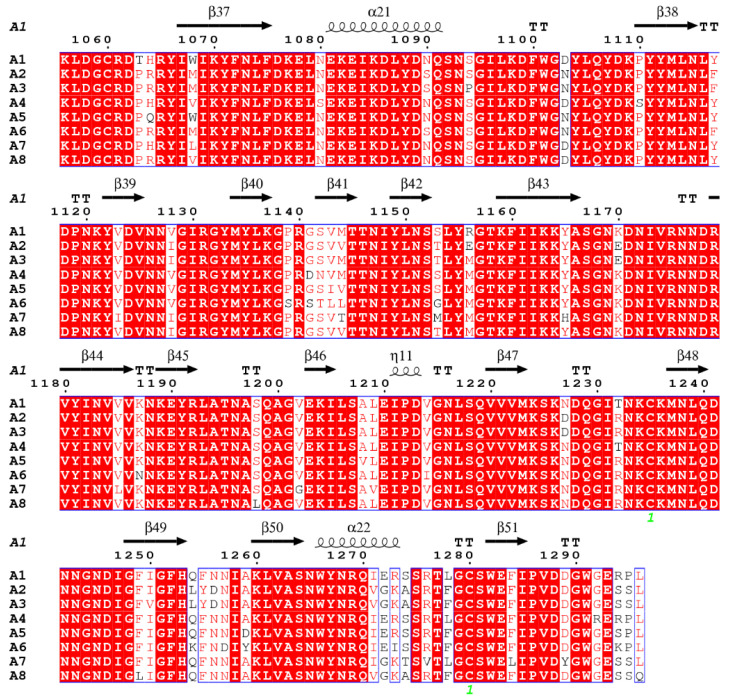
Sequence alignment of BoNT/A1 to /A8 cell-binding domains. Regions shown in white text with a red background indicate 100% identity. Regions/residues with high sequence similarity are shown in red text with a white background. Residues that lack both identity and similarity are shown in black text with a white background. Secondary structural elements are labelled α for α-helix, β for β-strand, and TT for turn. The residue numbering and secondary structural features are with respect to BoNT/A1 (3BTA), meaning the residue numbering for BoNT/A3 and /A4 is offset by +4 and -6, respectively. UniprotKB accession numbers in parentheses for the following BoNT/A subtypes: BoNT/A1 (P0DPI1), BoNT/A2 (Q45894), BoNT/A3 (D3IV24), BoNT/A4 (Q3LRX8), BoNT/A5 (C7BEA8), BoNT/A6 (C9WWY7), BoNT/A7 (K4LN57), and BoNT/A8 (A0A0A7PDB7).

These studies highlight the importance of determining precise differences in functionality across subtypes that arise due to minor changes in amino acid sequence. The H_C_ in particular, has been shown to be a ‘hotspot’ of this variation (Figure 3) [81], with the largest variation occurring within the N- and C-termini. X-ray crystallography is one strategy that may help deduce the cause of these functional differences.

**Figure 4:**
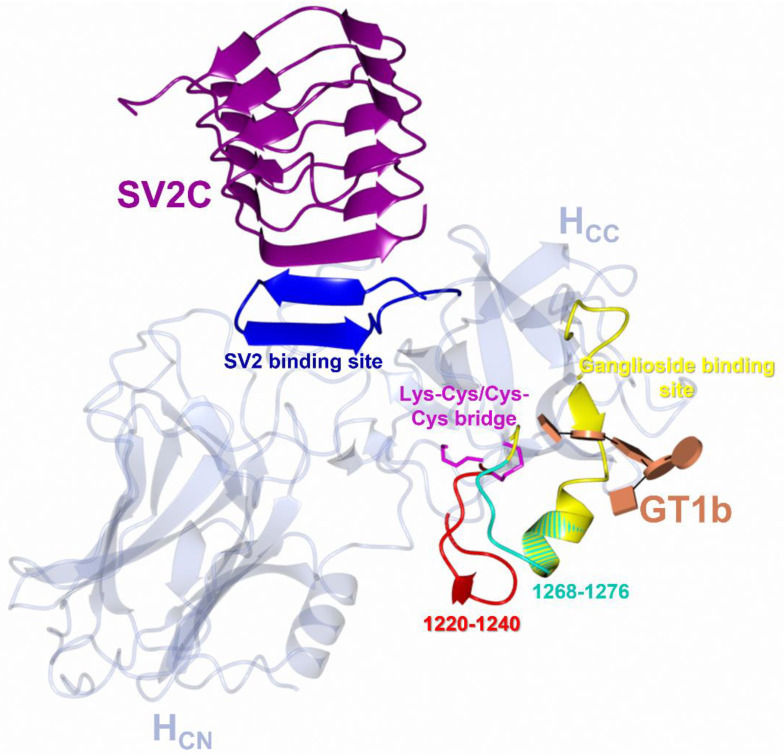
Features of BoNT/A cell-binding domain. The crystal structures of binding domain subtypes, H_C_/A1 to H_C_/A6, have allowed for the identification of six features of cell binding: The ganglioside binding site (yellow), SV2 binding site (blue), Lys–Cys/Cys–Cys bridge (magenta), two dynamic loop regions (1220-1240 (red) and 1268-1276 (cyan)), and a hinge rotation axis between the H_CN_ and H_CC_ domains. The structure of H_C_/A1 in complex with GT1b (PDB 2VU9) [90] and SV2 (PDB 5JLV) [37] is used here to show dual receptor binding and illustrate the features.

The wealth of structural data available on BoNT/A subtype cell-binding domains (H_C_/A1 to H_C_/A6) alone and in complex with their receptors have revealed features related to SV2 and ganglioside receptor binding [37,82,83,84,85,86,87,88,89,90,91,92]. In particular, the structure of H_C_/A1 in complex with human glycosylated SV2C [37] and of H_C_/A1 to H_C_/A6 in complex with the ganglioside GD1a (GM1b for H_C_/A5) [85,86,87,88,89,90] (Table 1) have identified six structural features that appear to be common to the cell-binding domain (Figure 4).

## 2. Cell-Binding Domain

### 2.1. Ganglioside Binding Site

**Figure 5:**
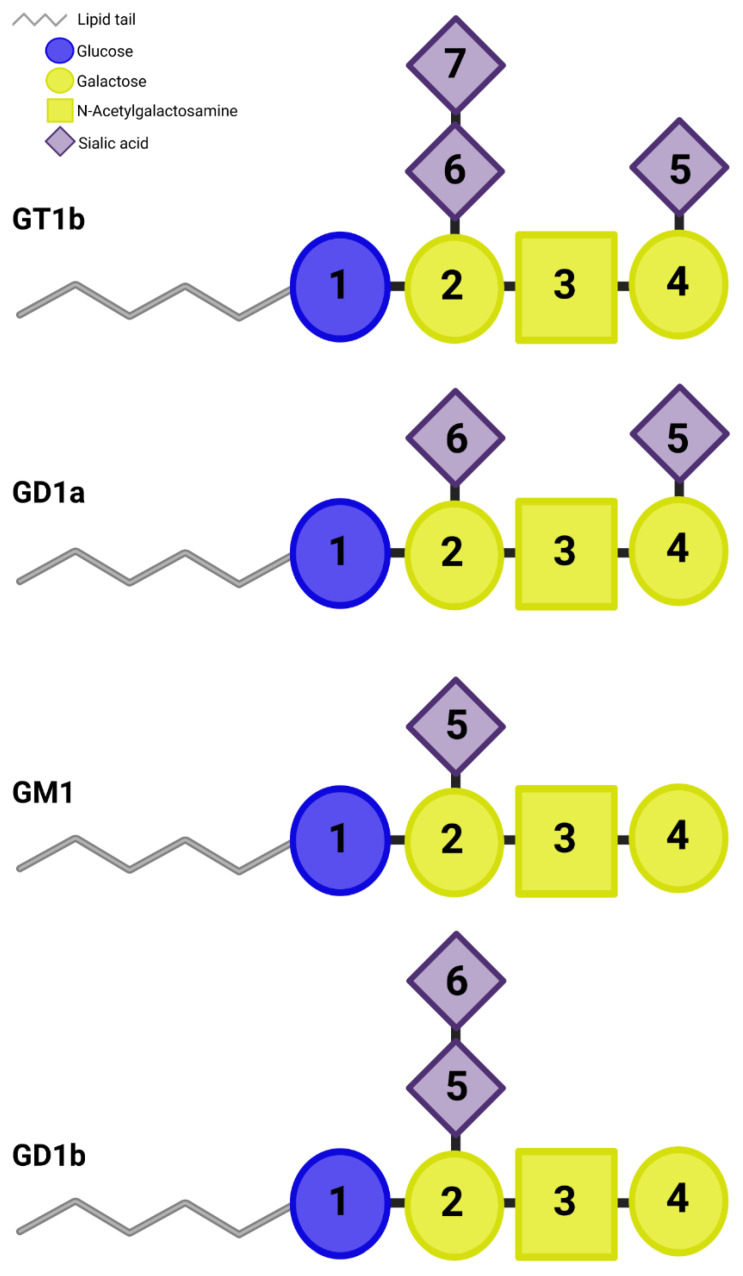
Common neuronal gangliosides. Gangliosides comprise a hydrophobic lipid tail and a hydrophilic sugar moiety (represented by 1–6). The schematic representation is of the most common neuronal gangliosides, GT1b, GD1a, GM1, and GD1b, illustrating the difference in sialic acid composition. Blue circle = glucose, yellow circle = galactose, yellow square = N-acetylglucosamine, purple diamond = sialic acid.

Gangliosides are ubiquitous within cell membranes, consisting of a membrane-bound lipid tail and an extracellular glycan ‘head-group’ [93]. Four gangliosides (GM1, GD1a, GT1b, and GD1b) (Figure 5) constitute 90% of all gangliosides in mammalian cells [94], and H_C_/A is known to bind both GT1b and GD1a [95]. 

**Figure 6:**
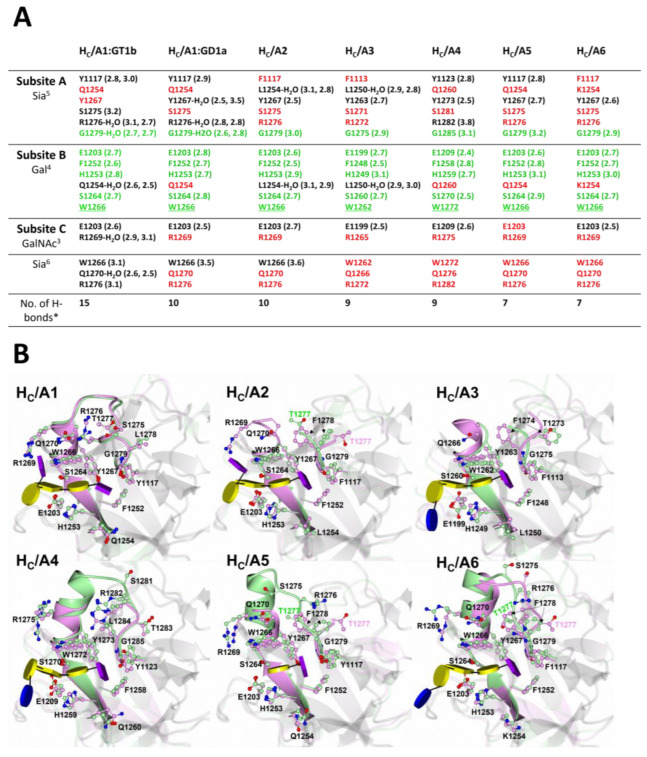
Ganglioside binding site of BoNT subtypes /A1 to /A6. (**A**) Table of ganglioside binding residues for H_C_/A1 through to H_C_/A6, with equivalent residues aligned. Green highlights binding residues that are observed across all subtypes, and red highlights non-binding residues. The underlined tryptophan indicates π-stacking interaction with the ganglioside rather than a hydrogen bond. Hydrogen bond distances (Å) are shown in parentheses, where for water-mediated interactions the first length is the protein-water distance and the second is the water-oligosaccharide distance. * The number of hydrogen bonds includes water-mediated interactions as a ‘single’ hydrogen bond. (**B**) Structural changes that occur at the amino acid level upon binding ganglioside (sugar moieties are as in Figure 5). The apo structures of each subtype are displayed in green and the ganglioside-bound structures in magenta. The arrows in H_C_/A2, H_C_/A3, H_C_/A5, and H_C_/A6 indicate a widening of the 1268–1276 loop and associated flip between residues 1277 (1273^A3^) and 1278 (1274^A3^) upon ganglioside binding. PDB codes are in parentheses for the following structures: H_C_/A1 (2VUA) [90], H_C_/A1:GT1b (2VU9) [90] H_C_/A1:GD1a (5TPC) [89], H_C_/A2 (7Z5T) [85], H_C_/A2:GD1a (7Z5S) [85], H_C_/A3 (6F0O) [82], H_C_/A3:GD1a (6THY) [86], H_C_/A4 (6F0P) [82], H_C_/A4:GD1a (7QPT) [87], H_C_/A5 (6TWP) [84], H_C_/A5:GM1b (7QPU) [87], H_C_/A6 (6TWO) [84], H_C_/A6:GD1a (8AGK) [88].

Binding occurs within a β-hairpin at the C-terminus of the H_CC_ subdomain (Figure 4 and Figure 6) [85,86,87,88,89,90], a region referred to as the ganglioside binding site (GBS), which is formed partly by a conserved ‘H…SxWY…G’ peptide motif [67] (Figure 3 and Figure 6). 

The H_C_/A1:GT1b, H_C_/A1:GD1a, H_C_/A2:GD1a, H_C_/A3:GD1a, H_C_/A4:GD1a, H_C_/A5:GM1b, and H_C_/A6:GD1a structures [85,86,87,88,89,90] (Table 1) have revealed the precise molecular interactions present across the H_C_/A:ganglioside interface. Six binding residues across the subtypes analysed here (H_C_/A1 to H_C_/A6) are conserved (Figure 6A), with some variation depending on the ganglioside. These residues are also conserved for H_C_/A7 and H_C_/A8, which suggests that they may also be involved with ganglioside binding (Figure 3) [83].

H_C_/A1 has been co-crystallised with both GT1b and GD1a, and these structures show a difference in binding residues (Figure 6A). Although the monosaccharides involved in binding are identical (Figure 5 and Figure 6A), H_C_/A1 has a higher affinity for GT1b than GD1a (GT1b > GD1a > GM1) [95]. Given that GT1b and GD1a differ by only one monosaccharide (Figure 5), the variability in binding has been proposed to be due to the additional sialic acid (Sia^7^) in GT1b enhancing the rigidity of Sia^6^ and altering the oligosaccharide torsion angles [89]. In addition, H_C_/A1 forms 15 hydrogen bonds (including water-mediated hydrogen bonds) with GT1b, but only 10 with GD1a [89,90] (Figure 6A). Following on from this, the relative affinity that each H_C_/A subtype has for GD1a may be inferred from the number of hydrogen bonds present across the interface, which suggests that H_C_/A1 and H_C_/A2 possess the highest affinity for GD1a (A1/A2 > A3/A4 > A6) [85,86,87,88,89]. Interestingly, two separate investigations of H_C_/A1 ganglioside binding showed variable affinity for GD1a—0.6 μM for the entire GD1a molecule [95] and 1 μM for the sugar moiety only [89]. This indicates that the lipid itself may contribute to the binding affinity of H_C_/A for gangliosides.

The GBS can be divided into three subsites ‘A’, ‘B’, and ‘C’ (Figure 6A) [89]. Subsite ‘A’ is occupied by Sia^5^ but the orientation of this sugar group within the subsite varies across the H_C_/A subtypes. In the H_C_/A2:GD1a, H_C_/A3:GD1a, H_C_/A4:GD1a, H_C_/A5:GM1b, and H_C_/A6:GD1a structures (Table 1), Sia^5^ is oriented perpendicular to Gal^4^, whereas in H_C_/A1:GD1a it is linear [85,86,87,88,89,90] (Figure 6B). This difference in Sia^5^ positioning can be attributed to several changes across the subtypes. H_C_/A1:GT1b forms five hydrogen bonds with sia^5^ [90], whereas H_C_/A1:GD1a [89] and H_C_/A4 [87] form four, H_C_/A2 [85], H_C_/A3 [86], and H_C_/A5 [87] form three, and H_C_/A6 forms two [88] (Figure 6A). Subsite ‘A’ also has amino acid variations at two sites corresponding to Tyr 1117 of H_C_/A1 (Tyr 1117^A5^, Tyr 1123^A4^, Phe 1117^A2/A6^, Phe 1113^A3^) and Gln 1254 of H_C_/A1 (Gln 1254^A5^, Gln 1260^A4^, Leu 1254^A2^, Leu 1250^A3^, Lys 1254^A6^) (Figure 3 and Figure 6A). The presence of a Tyr residue in the first site explains the higher hydrogen bond count for H_C_/A1, H_C_/A4, and H_C_/A5, because it forms a hydrogen bond with Sia^5^ through its side-chain hydroxyl group. The variation in hydrogen bonding to Arg 1276^A1/A2/A5/A6^/1272^A3^/1282^A4^, may also contribute to this difference in Sia^5^ orientation. In the H_C_/A2:GD1a, H_C_/A3:GD1a, H_C_/A5:GM1b, and H_C_/A6:GD1a structures (Table 1), this Arg residue does not contribute to hydrogen bonding, whereas for H_C_/A4:GD1a, the hydrogen bond involves the amine nitrogen atom, and for H_C_/A1:GD1a and H_C_/A1:GT1b, the hydrogen bond is via the carbonyl oxygen atom of Arg 1276 through a water-mediated interaction [85,86,87,88]. Considering that this Arg residue is conserved in all subtypes (Figure 3) but does not contribute to ganglioside binding for each subtype, ganglioside-binding residues cannot be inferred from amino acid sequence comparison alone.

Further to these specific amino acid variations, a dynamic loop region corresponding to H_C_/A1 residues 1268-1276 also contributes to differences in binding across the subtypes. This loop forms part of subsite ‘A’ and has the highest sequence variability within the GBS (Figure 3). It has been shown to widen upon ganglioside binding for H_C_/A2, H_C_/A3, H_C_/A5, and H_C_/A6 (Figure 6B, arrows), along with an associated flip at residues Thr 1277^A2/A5/A6^/1273^A3^ and Phe 1278^A2/A5/A6^/1274^A3^. The latter rotates towards the GBS and contributes to the formation of a hydrophobic pocket with Phe 1117^A2/A6^/1113^A3^ (Tyr 1117^A5^) and Phe 1252^A2^ (Figure 6B). The equivalent residue in H_C_/A1 and H_C_/A4 (Leu 1278^A1^/1284^A4^) does not flip; however, the position of this residue does differ between the subtypes. Prior to binding GD1a, Leu 1284^A4^ is already positioned within subsite ‘A’, contributing to the hydrophobic pocket, whereas for H_C_/A1, the Leu 1278^A1^ does not alter conformation upon binding GD1a and does not contribute to the hydrophobic pocket. Interestingly, in the H_C_/A1:GT1b structure Leu 1278^A1^ does flip towards the binding site [90]. Together, these differences demonstrate both the flexibility of the ganglioside moiety and variability of subsite ‘A’ across H_C_/A subtypes.

Five of the six totally conserved residues form subsite ‘B’ which is occupied by Gal^4^ upon ganglioside binding (Figure 6A). Gal^4^ is sandwiched between His 1253^A1/A2/A5/A6^/1249^A3^/1259^A4^ and Trp 1266^A1/A2/A5/A6^/1262^A3^/1272^A4^ forming π-stacking interactions, and hydrogen bonds to Glu 1203^A1/A2/A5/A6^/1199^A3^/1209^A4^, Phe 1252^A1/A2/A5/A6^/1248^A3^/1258^A4^, His 1253^A1/A2/A5/A6^/1249^A3^/1259^A4^, and Ser 1264^A1/A2/A5/A6^/1260^A3^/1270^A4^ (Figure 6B). An additional hydrogen bond to Gal^4^ is observed in the H_C_/A1:GT1b, H_C_/A2:GD1a, and H_C_/A3:GD1a structures through a water-mediated interaction with Gln 1254^A1^ (Leu 1254^A2^/1250^A3^). This large number of hydrogen bonding with its sugar group and low average B-factor across all H_C_/A:ganglioside structures [85,86,87,88,89,90] makes subsite ‘B’ the most stable and tightly bound pocket for a ganglioside monosaccharide. Together, subsites ‘A’ and ‘B’ appear to provide high specificity for glycans containing a terminal Sia–Gal moiety [89].

Subsite ‘C’ binds GalNAc3, forming one hydrogen bond via Glu 1203^A1/A2/A5/A6^/1199^A3^/1209^A4^ in the GD1a bound structures, and an additional water-mediated interaction in the H_C_/A1:GT1b structure (Figure 6A). The monosaccharide, Sia^6^, does not occupy a subsite but it does form a weak hydrogen bond with Trp 1266 in the H_C_/A1:GD1a/GT1b and H_C_/A2:GD1a structures. It is possible that this interaction occurs for binding sites H_C_/A3 to H_C_/A6 but has not been observed in the crystal structures because of the weak nature of the indole–hydrogen bond [96].

### 2.2. SV2 Binding Site

Synaptic vesicle glycoprotein 2 isoforms (SV2A, B, and C) are a small family of secretory vesicle glycoproteins present in all synapses, they consist of 12 transmembrane helices and a large extracellular domain with three *N*-linked glycosylation sites [97]. SV2 proteins are multifunctional as they are involved in the modulation of exocytosis and are essential for normal function of the nervous and endocrine systems [98]. Overexpression of SV2A and SC2C in insulin-secreting cells reduces glucose-induced secretion [99], whereas loss of SV2B results in a reduction in neurotransmission [100].

**Figure 7:**
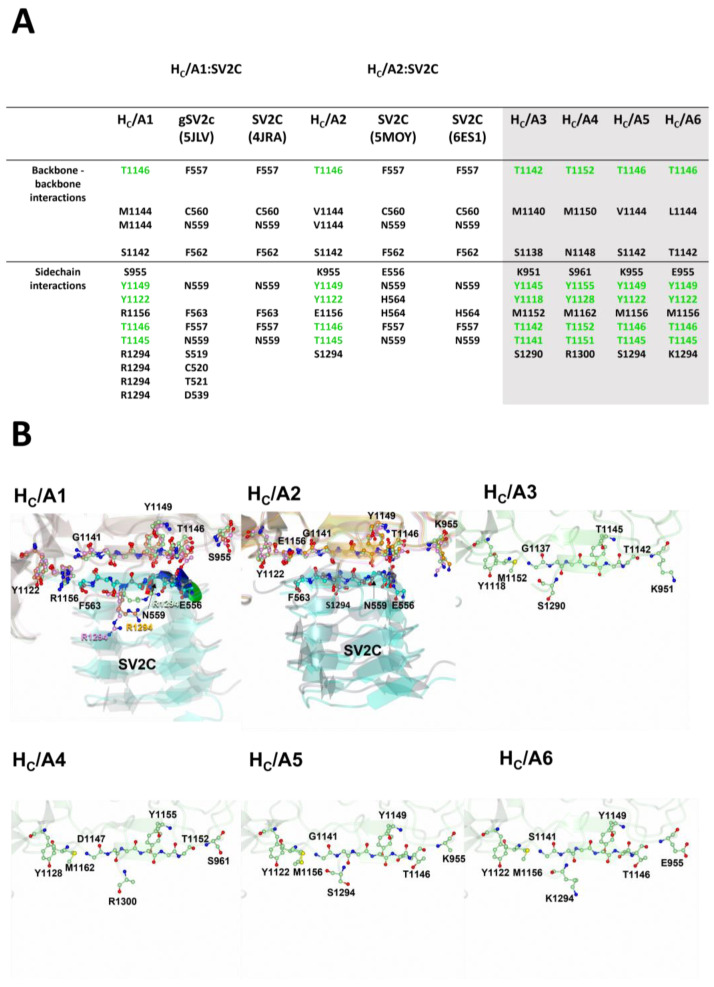
SV2 protein-moiety binding site. (**A**) Table listing the SV2 residues that interact with the binding site in H_C_/A1 and H_C_/A2. Interacting residues are aligned. The equivalent residues in H_C_/A3 to H_C_/A6 are also shown to highlight a putative SV2 binding site (conserved residues are displayed in green). (**B**) Structural differences of all interacting residues (ball and stick) between H_C_/A1, and H_C_/A2, the apo structures are shown in green (H_C_/A1 [PDB 2VUA] and H_C_/A2 [PDB 7Z5T]), and the SV2-bound structures in pink (H_C_/A1:gSV2C (PDB 5JLV) and H_C_/A1:SV2C [PDB 5MOY]) and orange (H_C_/A1:SV2 [PDB 4JRA], and H_C_/A2 [PDB 6ES1]). Putative SV2 protein-moiety binding site of H_C_/A3 to H_C_/A6 are also shown. PDB codes are in parentheses for the following structures: H_C_/A1 (2VUA) [90], H_C_/A1:gSV2C (5JLV) [37], H_C_/A2 (7Z5T) [85], H_C_/A2:SV2C (5MOY/6ES1) [91,92], H_C_/A3 (6F0O) [82], H_C_/A4 (6F0P) [82], H_C_/A5 (6TWP) [84], and H_C_/A6 (6TWO) [84].

**Figure 8:**
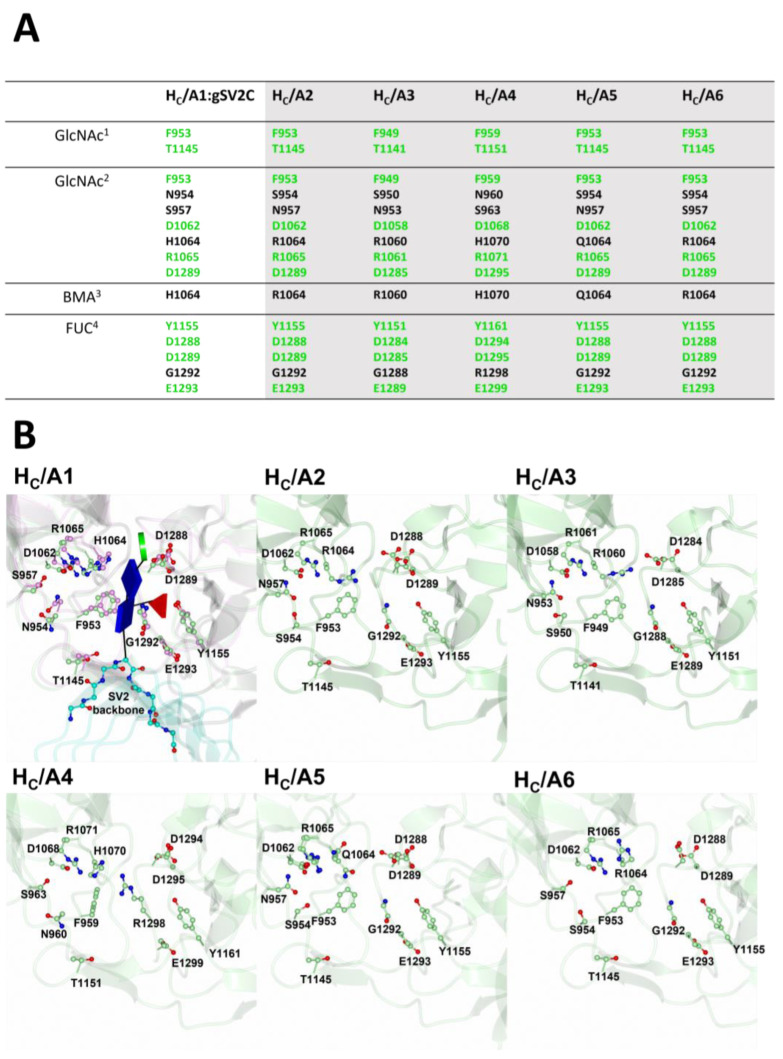
SV2 glycan-moiety binding site. (**A**) Table listing the H_C_/A1 glycan-binding residues, and equivalent residues in H_C_/A2 to H_C_/A6. Conserved residues are shown in green. (**B**) The SV2 glycan binding sites of H_C_/A1, and equivalent for H_C_/A2 to H_C_/A6. For H_C_/A1 the unbound (green) and SV2 bound (magenta) structures are overlayed. PDB codes are in parentheses for the following structures: H_C_/A1 (2VUA) [90], H_C_/A1:gSV2C (5JLV) [37], H_C_/A2 (7Z5T) [85], H_C_/A2:SV2C (5MOY/6ES1) [91,92], H_C_/A3 (6F0O) [82], H_C_/A4 (6F0P) [82], H_C_/A5 (6TWP) [84], and H_C_/A6 (6TWO) [84].

BoNT/A can bind to the extracellular domain of all three isoforms of SV2 (SV2A, SV2B, and SV2C), recognising both the protein (Figure 7) and glycan moiety [41] (Figure 8). H_C_/A has the highest affinity for SV2C [101], and binding of H_C_/A1 and H_C_/A2 to the protein moiety has been characterised through X-ray crystallography [37,91,92,102]. Three residues of H_C_/A1 and H_C_/A2 (1142, 1144, and 1146) contribute to an extended β-sheet stacking interaction with the SV2C protein moiety (Phe 557, Asn 559, Cys 560, and Phe 562) (Figure 7A) [37,91,92,102]. Considering that these residues are not conserved across the H_C_/A subtypes, (Figure 3 and Figure 7A), they are unlikely to contribute to any significant difference in SV2 binding. There are, however, two highly conserved residues in all H_C_/A subtypes (Thr 1145^A1/A2/A5/A6^/1141^A3^/1151^A4^ and Thr 1146^A1/A2/A5/A6^/1142^A3^/1152^A4^), that are essential for H_C_/A1- and H_C_/A2-binding of the SV2C protein moiety via their side-chain atoms (Figure 7B) [102]. Away from the extended β-sheet stacking interaction, there are additional residues that form hydrogen bonds through their side-chain atoms, and some of these residues are conserved across the other subtypes (Figure 3 and Figure 7A). In H_C_/A1, Arg 1156^A1^ forms a unique π-stacking interaction with Phe 563 of SVC, which has been shown to contribute to SV2C binding affinity [102]. In H_C_/A2:SV2C, Arg 1156^A1^ is substituted by Glu 1156^A2^, which forms a hydrogen bond with His 564 instead (Figure 7B). However, these few and varying interactions that H_C_/A1 and H_C_/A2 have with the SV2C protein moiety do not explain their high affinity and specificity for SV2 [37,102].

The glycan-moiety binding site is crucial for high affinity binding of BoNT/A for SV2, because binding required glycosylated SV2A/B and enhanced binding for glycosylated SV2C [37,41]. The crystal structure of the BoNT/A1 binding domain in complex with glycosylated human SV2C (H_C_/A1:gSV2C) revealed a high number of interactions with an *N*-linked glycan on Asn-559 of gSV2C [37] (Figure 8A). This residue is conserved in all vertebrate SV2-homologs. Two key residues involved in H_C_/A1 glycan binding are Phe 953 and His 1064, which form π-stacking interactions with two GlcNAc molecules of the Asn 559 glycan (Figure 8B). A further 12 residues (Phe 953, Asn 954, Ser 957, Asp 1062, His 1064, Arg 1065, Thr 1145, Tyr 1155, Asp 1288, Asp 1289, Gly 1292, Glu 1293) form a network of hydrogen bonds with the glycan through water-mediated interactions (Figure 8A). 

Most of these interacting residues are conserved across the subtypes with the exception of Asn 954, Ser 957, His 1064, and Gly 1292 (Figure 3 and Figure 8). These differences in residues at the glycan binding site may alter binding and contribute to variation in affinity across BoNT/A subtypes. In particular, the replacement of His 1064^A1^ with an arginine in H_C_/A2, H_C_/A3, H_C_/A6, and H_C_/A8 (Arg1064^A2/A6/A8^/1060^A3^) makes them unable to form a *π*-stacking interaction with one of the GlcNAc molecules.

Although the binding site of H_C_/A2 is fairly rigid, two different structures of H_C_/A2:SV2C revealed alternative ways of binding to the SV2C protein moiety (Figure 7B) [91,92]. This is likely due to the interaction involving predominantly main chain backbone atoms of H_C_/A2. Whether this plasticity is due to the lack of glycosylation remains unclear. It is possible that glycosylated Asn 559 may restrict the interaction that H_C_/A subtypes have with the SV2 protein moiety, therefore, non-glycosylated forms of SV2 may sometimes be misleading in how H_C_/A binds. In order to obtain a full structural understanding of receptor binding across all BoNT/A subtypes, detailed structures of H_C_/A2 through to H_C_/A8 in complex with glycosylated SV2 are required. 

### 2.3. The Hinge Region

**Figure 9:**
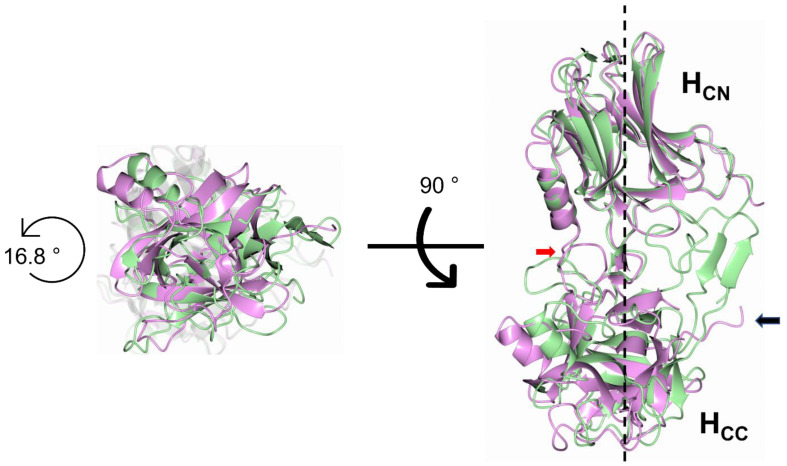
Hinge motion of H_CN_ and H_CC_ subdomains. Two crystal forms of H_C_/A6 revealed a 16.8° hinge motion between the H_CN_ and H_CC_ subdomains [88]. The H_CN_ from both structures were aligned independently to highlight the difference in H_CC_ orientation between H_C_/A6 crystal form I (green) (PDB 6TWO) [84] and H_C_/A6 crystal form II (magenta) (PDB 8ALP) [88]. The image on the left is orientated perpendicular to the rotation axis, with the image on the right a view at 90°. For the image on the right, the rotation axis is shown by the dotted line. The hinge residues are indicated by the red arrow and the difference in SV2 binding site-loop positioning between crystal form I (fully modelled) and II (partially modelled) is shown by the black arrow.

A comparison of the H_C_/A1 to H_C_/A6 structures to their ganglioside-bound structures [82,83,84,85,86,87,88,89,90] revealed a subtle hinge rotation between the H_CN_ and H_CC_ subdomains. Additionally, a new crystal form (crystal form II) of H_C_/A6 [88] revealed a much larger rotation of ~16.8° (Figure 9), which appears to prevent the formation of a β-stacking interaction between a portion of the H_CN_ subdomain and the SV2 binding site of the H_CC_ subdomain. 

This suggested that the flexibility of the SV2 binding site increased upon rotation, allowing it to protrude out towards the surface of the protein (Figure 9, black arrow) [88]. Although a large hinge rotation has only been observed in H_C_/A6, such a feature may exist across the subtypes [85,87,88]. The function of the hinge rotation is yet to be established, but it may contribute to the enhancement of SV2 binding or translocation. Upon binding to the dual receptor complex (Figure 1B), both the LC and H_N_ need to reposition to be in contact with the membrane to initiate translocation [36,49,51,90,103,104,105]. Additionally, the ‘BoNT-switch’, which is a stretch of 47 residues that alters conformation from an α-helix to a β-finger at acidic pH, would need to reorientate to come into contact with the cell membrane [106]. The hinge may therefore offer a region of flexibility that could accommodate this repositioning. Further structural studies of the toxin in the presence of a membrane may aid our understanding of the function of this hinge motion. Other than the new crystal form of H_C_/A6, crystallographic and in-solution structural studies of BoNT have revealed very little motion within the molecule [107]. In full-length structures of BoNT this is perhaps due to the regulatory function that each domain exhibits on one another, for example, the H_C_ has been shown to reduce the pH range in which the H_N_ domain forms channels in phospholipid bilayers [54,108], and the H_N_ moderates the LCs catalytic activity [33]. It has been established that a change in pH alone is not sufficient to elicit a conformational change within the BoNT/B [109], and if this holds true for BoNT/A, elucidating binding of BoNT with its dual receptor complex at low pH may reveal more biologically relevant conformational changes.

### 2.4. Lys–Cys/Cys–Cys Bridge

Between the Nζ of Lys 1236 and the Sγ of Cys 1280 in H_C_/A5, a continuation of electron density was observed, characteristic of a Lys–Cys bridge between the two atoms [84]. Similar findings were later observed in the crystal structures of H_C_/A2 [85] and H_C_/A6 [88]. A recent meta-analysis of structures deposited with the protein data bank revealed a large number of unreported Lys–O–Cys (Nζ-O-Sγ, NOS) bridges [110]; the first of which was identified in a transaldolase enzyme from *Neisseria gonorrhoeae* that demonstrated a redox-dependent ‘switch’-like nature [111]. Consequently, the bridging interaction in H_C_/A2 and H_C_/A6 (crystal form II) was characterised as a NOS bridge due to the apparent redox-dependency in the available crystal structures of H_C_/A2 [85] (Table 1). Although sub-angstrom resolution data are required to unequivocally determine the identity of the bridging atom [112], the current reported literature indicates that ‘O’ is the most probable connecting atom [110,111]. Synchrotron radiation (generally used for X-ray data collection in the crystal structure determination process) is known for its reducing properties, therefore, the presence of NOS bridges in H_C_/A subtypes is unlikely to be an artefact of data collection [111]. In fact, the opposite is true, radiation damage is likely to break NOS bridges [110].

**Figure 10:**
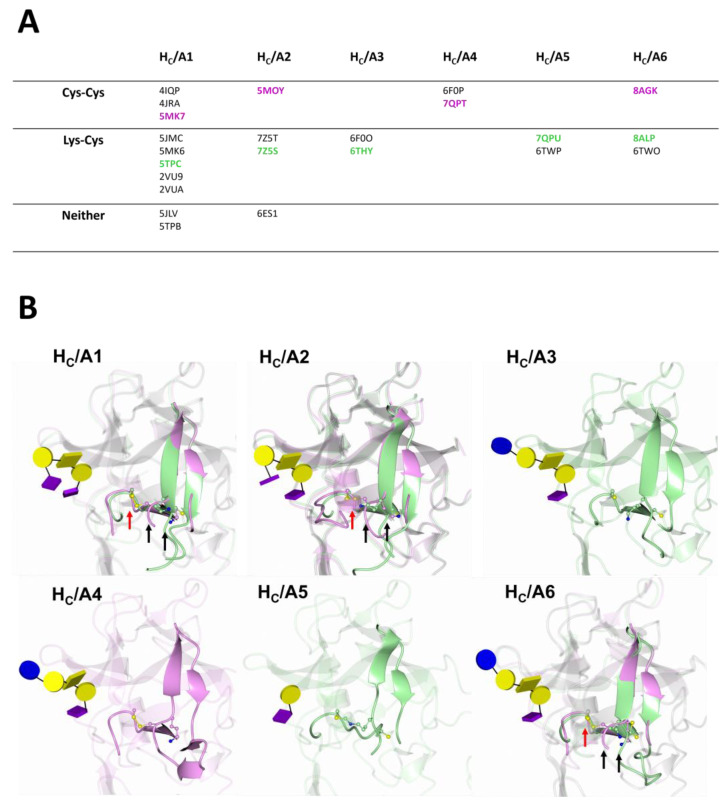
The Lys–Cys/Cys–Cys bridge and associated structural change. (**A**) Table of available crystal structures of H_C_/A subtypes categorised by the possible presence of Lys 1236^A1/A2/A5/A6^/1232^A3^/1242^A4^-Cys 1280^A1/A2/A5/A6^/1276^A3^/1286^A4^, Cys 1280^A1/A2/A5/A6^/1276^A3^/1286^A4^-Cys 1235^A1/A2/A5/A6^/1231^A3^/1241^A4^, or no bridge at all. Structures possessing a Lys–Cys bridge were observed in the deposited coordinates for H_C_/A2 (7ZSQ/7Z5S), H_C_/A5 (6TWP) and H_C_/A6 (8ALP), whereas the other remaining structures were re-analysed and reported to show electron density between the Lys–Cys residue and structural changes consistent with the Lys–Cys bridge. (**B**) Structural comparison of loops 1220-1240^A1/A2/A5/A6^ (1216-1236^A3^ and 1226-1246^A4^) and 1268-1276^A1/A2/A5/A6^ (1264-1272^A3^ and 1274-1282^A4^) associated with occurrence of either a Lys–Cys (green) or Cys–Cys (magenta) bridge. A Cys–Cys bridge appears to ‘pull’ the 1220-1240^A1/A2/A5/A6^ loop (1216-1236^A3^ and 1226-1246^A4^) (black arrows) towards the 1268-1276^A1/A2/A5/A6^ loop (1264-1272^A3^ and 1274-1282^A4^) (red arrow) and alter the direction of the β-hairpin slightly. The position of bound ganglioside for each subtype is shown to illustrate the proximity of the Lys–Cys/Cys–Cys bridge to the GBS (sugar moieties are as in Figure 5). The structures used to illustrate structural differences in the presence of either a Lys–Cys or Cys–Cys bridge are highlighted in Figure 10A: H_C_/A1 Lys–Cys (5TPC) [89], H_C_/A1 Cys–Cys (5MK7) [83], H_C_/A2 Lys–Cys (7Z5S) [85], H_C_/A2 Cys–Cys (5MOY) [92], H_C_/A3 Lys–Cys (6THY) [86], H_C_/A4 Cys–Cys (7QPT) [87], H_C_/A5 Lys–Cys (7QPU) [87], H_C_/A6 Lys–Cys (8ALP) [88], H_C_/A6 Cys–Cys (8AGK) [88].

It is theoretically possible for all H_C_/A subtypes to form a NOS bridge because both Lys 1236^A1/A2/A5/A6^/1232^A3^/1242^A4^ and Cys 1280^A1/A2/A5/A5/A6^/1276^A3^/1286^A4^ are conserved (Figure 3). To check if the NOS bridge has gone previously undetected in BoNT/A structures, we reviewed the presence of the NOS bridge in all the available crystal structures of H_C_/A subtypes (Table 1). If we consider that a continuation of electron density between Lys 1236^A1/A2/A5/A6^/1232^A3^/1242^A4^) and Cys 1280^A1/A2/A5/A6^/1276^A3^/1286^A4^ is indicative of a NOS bridge, the NOS bridge would appear to be the most common interaction (Figure 10A). This NOS bridge is likely to be dynamic because the Cys 1280^A1/A2/A5/A6^/1276^A3^/1286^A4^ residue may also form a disulphide bond with Cys 1235^A1/A2/A5/A6^/1231^A3^/1241^A4^ or not form a bridge at all (Figure 10B). In total, 57% of H_C_/A structures show a potential NOS bridge, 35% a Cys–Cys bridge, and 8.0% no bridge at all (Figure 10A). Interestingly, two crystal forms of H_C_/A1 were obtained from the same crystallisation condition [83]. One showed a Cys–Cys bridge and the other no bridge [83], indicating that the crystallisation condition does not dictate which interaction is observed in the crystal structure. The location of this bridging interaction is close to the GBS and connects two flexible regions of the molecule: the ‘dynamic loop’ (1268-1276) within subsite ‘A’, and a β-hairpin at residues 1220-1240 (Figure 10B). This β-hairpin adopts different conformations across the subtypes depending on whether a NOS or a Cys–Cys bridge is present (Figure 10B). This difference is likely due to the different lengths of the bridging interaction—a Cys–Cys bridge appears to ‘pull’ the β-hairpin loop closer to the dynamic loop of subsite ‘A’ (Figure 10B). Residues corresponding to Lys 1236^A1^, Cys 1235^A1^, and Cys 1280^A1^ are conserved across the BoNT/A subtypes (Figure 3); however, only the equivalent Cys residues are conserved across other serotypes (BoNT/B, /E, /F, and /G) [83], that have been shown to affect humans. The NOS/Cys–Cys bridge, therefore, appears to be unique to all BoNTs with a H_C_/A binding domain. The function of the NOS/Cys–Cys bridge in BoNT/A is unknown, but its existence close to the GBS suggests it may have a biological function.

## 3. Conclusions and Future Perspectives

The structural data of BoNT/A1 to /A6 subtype cell-binding domains (H_C_/A1 to H_C_/A6) have provided a wealth of information, not only on subtype-to-subtype variability, but also on how the cell-binding domain recognises its receptors and the implication of this on functional features. The crystal structures have allowed for characterisation of six features of cell binding: The ganglioside binding site (GBS), a dynamic loop, a possible NOS/Cys–Cys bridge, the SV2 protein and glycan binding sites, and the H_CN_–H_CC_ subdomain hinge. The GBS consists of three subsites (‘A’, ‘B’, and ‘C’) that are able to accommodate gangliosides containing a terminal Sia–Gal–GalNAc moiety, with preference for GT1b and GD1a. A dynamic loop forms part of subsite ‘A’ and shows variation across the subtypes, appearing to widen upon GD1a (GM1b for H_C_/A5) binding in H_C_/A2, H_C_/A3, H_C_/A5, and H_C_/A6. Close to the GBS is a possible alternating bridging interaction unique to BoNT/A that switches between Lys–Cys and Cys–Cys, with some evidence of redox-dependency. The two cysteines are conserved across mammalian-targeting BoNT serotypes (BoNT/A, /B, /E, and /F), which may indicate a functional role in the mechanism of intoxication. Further experimental investigation is needed to establish if there is a definitive biological role.

BoNT/A recognises its protein receptor (SV2) through both a protein and glycan moiety, with the latter portion appearing to contribute significantly to the high specificity and affinity of binding observed across BoNT/A subtypes. Each of these features is located within the H_CC_ subdomain, whereas the function of the H_CN_ still remains unclear. There appears to be a rotation axis between the two subdomains that might aid in SV2 binding or subsequent translocation. We highlight the importance of more biologically relevant structural data, in particular with reference to non-glycosylated SV2-bound structures that show limited information regarding the true nature of binding. With newly emerging serotypes and subtypes, the expansion of the available atomic data on receptor binding among BoNTs would prove beneficial for both the development of antibodies against BoNT, and the engineering of BoNT molecules for therapeutic purposes. 

## Figures and Tables

**Table 1:** List of BoNT/A subtype cell-binding domains and associated RCSB PDB codes. A comprehensive analysis of the structures listed here forms the basis of this review.

Structure	PDB Code	Resolution
H_C_/A1	2VUA	1.70 Å
H_C_/A1:GT1b	2VU9	1.60 Å
H_C_/A1:GD1a	5TPC	2.00 Å
H_C_/A1:gSV2C	5JLV	2.00 Å
H_C_/A1:SV2C	4JRA	2.30 Å
H_C_/A2	7Z5T	1.63 Å
H_C_/A2:GD1a	7Z5S	2.10 Å
H_C_/A2:SV2	6ES1	2.00 Å
H_C_/A2:SV2	5MOY	2.30 Å
H_C_/A3	6F0O	1.60 Å
H_C_/A3:GD1a	6THY	1.75 Å
H_C_/A4	6F0P	1.34 Å
H_C_/A4:GD1a	7QPT	2.30 Å
H_C_/A5	6TWP	1.15 Å
H_C_/A5:GM1b	7QPU	2.40 Å
H_C_/A6 (crystal form I)	6TWO	1.35 Å
H_C_/A6 (crystal form II)	8ALP	1.50 Å
H_C_/A6:GD1a	8AGK	1.50 Å

## Data Availability

Not applicable.

## Abbreviations

ACh	Acetylcholine
BoNT/x	Botulinum neurotoxin (/serotype)
Gal	Galactose
GalNAc	N-acetylglucosamine
GBS	Ganglioside binding site
Glc	Glucose
HC/x	Heavy chain of botulinum neurotoxin (/serotype or /subtype)
H_C_/x	Cell-binding domain of botulinum neurotoxin (/serotype or /subtype)
H_CC_/x	C-terminal cell-binding subdomain of botulinum neurotoxin (/serotype or /subtype)
H_CN_/x	N-terminal cell-binding subdomain of botulinum neurotoxin (/serotype or /subtype)
H_N_/x	Translocation domain of botulinum neurotoxin (/serotype or /subtype)
LC/x	Light chain of botulinum neurotoxin (/serotype or /subtype)
NMJ	Neuromuscular junction
Sia	Sialic acid
SNAP-25	Synaptosomal-associated protein of 25 kDa
SNARE	Soluble *N*-ethylmaleimide-sensitive factor attachment protein receptor
SV2x	Synaptic vesicle glycoprotein 2 (x = A/B/C)
Syt I/II	Synaptotagmin (isoform I/II)
Trx	Thioredoxin
TrxR	Thioredoxin reductase
VAMP	Vesicle-associated membrane protein
